# Impact of Magnesium Stearate Presence and Variability on Drug Apparent Solubility Based on Drug Physicochemical Properties

**DOI:** 10.1208/s12248-020-00449-w

**Published:** 2020-05-21

**Authors:** P. Zarmpi, T. Flanagan, E. Meehan, J. Mann, Nikoletta Fotaki

**Affiliations:** 1 Department of Pharmacy and Pharmacology, University of Bath, Bath, BA2 7AY UK; 2 Pharmaceutical Technology & Development, AstraZeneca, Macclesfield, UK; 3 UCB Pharma, Chemin du Foriest, B-1420 Braine-l’Alleud, Belgium

**Keywords:** drug solubility, excipient variability, magnesium stearate, multivariate data analysis, physicochemical properties

## Abstract

Excipients are major components of oral solid dosage forms, and changes in their critical material attributes (excipient variability) and/or amount (excipient variation) in pharmaceutical formulations may present a challenge for product performance. Understanding the biopharmaceutical factors affecting excipient performance is recommended for the successful implementation of excipient variability on Quality by Design (QbD) approaches. The current study investigated the impact of magnesium stearate (MgSt) variability on the apparent solubility of drugs with a wide range of physicochemical properties (drug ionization, drug lipophilicity, drug aqueous solubility). Compendial and biorelevant media were used to assess the role of gastrointestinal (GI) conditions on the excipient effects on drug apparent solubility. The lipophilic nature of MgSt decreased the apparent solubility of most compounds. The reduction in drug apparent solubility was more pronounced for highly soluble and/or highly ionized drugs and in presence of more highly crystalline or smaller particle size MgSt. The use of multivariate data analysis revealed the critical physicochemical and biopharmaceutical factors and the complex nature of excipient variability on the reduction in drug apparent solubility. The construction of a roadmap combining drug, excipient and medium characteristics allowed the identification of the cases where the presence of excipient or excipient variability may present risks for oral drug performance.

**Electronic supplementary material:**

The online version of this article (10.1208/s12248-020-00449-w) contains supplementary material, which is available to authorized users.

## INTRODUCTION

Excipient variability (changes in material properties) and variation (changes in amount) can affect final product quality leading to batch failures, altered bioavailability and bioinequivalence within products [1, 2]. The need for successful implementation of excipient variability in Quality by Design (QbD) approaches is widely recognized. Excipients may present challenges in a biopharmaceutical perspective as their presence may impact drug stability, solubility, permeability and overall product performance [3]. Gastrointestinal factors may affect or be affected by excipient performance in pharmaceutical formulations [4]. Varying the critical material attributes of certain excipients may be problematic for oral drug absorption. Understanding the biopharmaceutical risks of excipient variability would be beneficial towards the development of robust oral solid dosage forms.

**Fig. 1:**
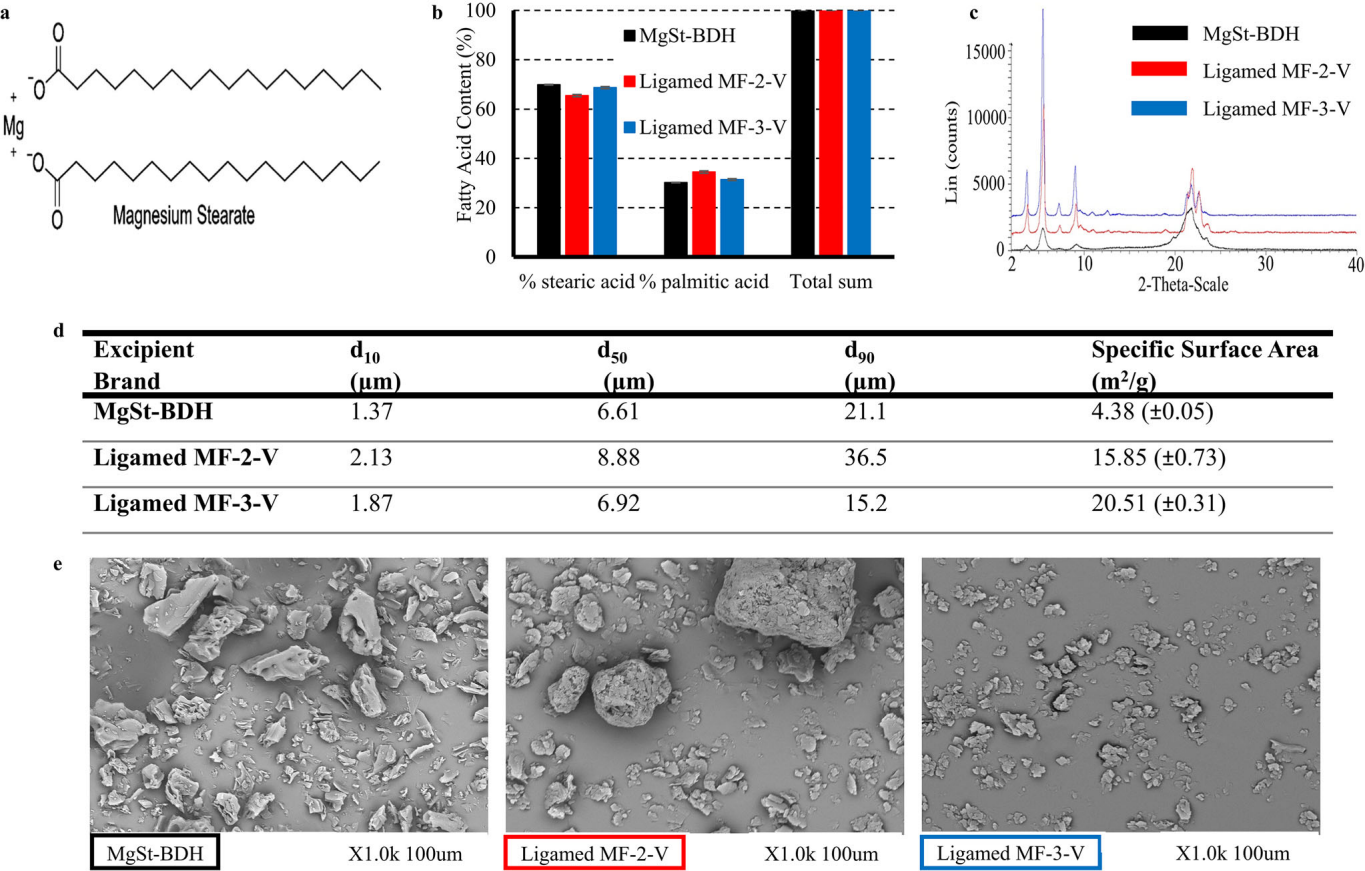
**a** Chemical structure of magnesium Stearate (ChemDraw Professional 15.0). **b** Relative content (%) and total sum (%) of stearic and palmitic acid. **c** XRPD patterns. **d** Particle size distribution and specific surface area. **e** SEM micrographs of the studied brands of MgSt. The different brands are shown as for (i) MgSt - BDH (black colour), (ii) Ligamed MF-2-V (red colour) and (iii) Ligamed MF-3-V (blue colour)

Magnesium stearate (MgSt) is a frequently used lubricant in tablet manufacturing due to its excellent properties [5]. Typical excipient levels used in immediate-release formulations range between 0.25–5% w/w [6]. MgSt can compromise drug release, as it acts as water repellent [7, 8] through the formation of a hydrophobic film around drug particles [9, 10]. MgSt may adhere to metal or powder/granule surfaces by its polar Mg^2+^ [5, 11] while the non-polar fatty acid orients away from the coated surface and is responsible for the negative impact on product performance [11] (Fig. 1a). Formation of mono- or multi-particulate layers around particles by the non-polar head of MgSt [12, 13] has also been suggested as a coating approach for solid dosage forms.

MgSt is a mixture of stearic and palmitic salts [4]. Several different hydrates and corresponding solid-state forms of MgSt have been identified: anhydrous, monohydrate (needle—shape or disordered), dihydrate (plate—shape), trihydrate (needle—shape) [14, 15]. MgSt samples may comprise of crystalline (single or combination of 2 or more forms) or disordered forms [15]. Molecular, structural, particle properties and excipient level have been identified as potential critical material attributes of MgSt [4]. The purity of MgSt (ratio of stearic and palmitic acid) may affect its lubrication efficiency. The delay in the dissolution of a highly soluble drug (paracetamol, United States Pharmacopeia (USP) 2 apparatus, 0.1 N HCl pH 1, 25 rpm, 37°C) was more pronounced in the presence of commercial MgSt (pharmaceutical grade mixture of stearic and palmitic salts with not less than 40% of stearic acid) compared with high purity MgSt (> 90% of stearic acid) [9]. The slower drug dissolution was attributed to the formation of an extensive hydrophobic layer around particles by commercial compared with high purity MgSt. The formation of the hydrophobic layer around particles by MgSt depends on the solid-state form of the excipient. Disordered MgSt hinders the formation of a smooth film around particles due to its irregular shape [16]. Milling crystalline MgSt breaks its crystal structure leading to less crystalline material which exhibits poor lubrication efficiency [17]. The significance of the degree of crystallinity (CRS) is highlighted as the negative effects of disordered MgSt on lubrication can dominate the lubrication efficiency of the crystalline portion [18]. Coating strength and uniformity depend on the particle size distribution (PSD) of the lubricant. Smaller particles create a thin but homogenous layer due to higher adhesion force on surfaces while larger particles have relatively less tendency to adhere on surfaces creating less uniform coats [4]. A statistical evaluation of the factors affecting the lubrication efficiency of MgSt identified that for crystalline MgSt, moisture content, Hausner ratio, PSD (d_50_) and specific surface area (SSA) were critical material properties [18]. Finally, the level of excipient in solid dosage forms may affect the coating efficiency of MgSt and its impact on product performance. Pronounced delay in tablet disintegration and drug dissolution when increasing the amount of MgSt on formulations has been reported as a result of decreased tablet wettability [10].

The biopharmaceutical factors affecting the impact of MgSt on product performance are not fully investigated. The extent to which MgSt delayed drug dissolution was found to depend on the solubility of the active pharmaceutical ingredient (API). Dissolution studies (USP 2 apparatus, 0.1 N HCl pH 1, 75 rpm, 37°C) of capsules containing drugs of different solubility showed that although MgSt presence delayed drug dissolution, the impact was more pronounced for the drug with the lowest solubility in the dissolution medium [7]. The pH of the solution will affect the performance of MgSt due to the ionization pattern of the excipient. The dissolution of metformin hydrochloride (USP 2 apparatus, 50 rpm, 37°C) from tablets containing MgSt was slower in acidic (simulated gastric fluid without pepsin (SGF) pH 1.2) compared with basic conditions (phosphate buffer pH 6.8) [19]. This difference in drug dissolution by MgSt was attributed to the generation of stearic acid in acidic media which causes a more pronounced delay on drug dissolution due to its lower solubility compared with the salt form [19].

The aim of this study was to identify the impact and criticality of MgSt variability and variation on drug apparent solubility from a biopharmaceutical perspective. MgSt variability and variation were assessed by selecting and characterizing three MgSt brands of different grades and/or suppliers to investigate the role of certain critical material attributes including purity (% of stearic and palmitic acid), CRS (intensity of diffractograms), PSD (d_10,_ d_50,_ d_90_) and excipient level (low: 2% w/w, high: 5% w/w) on drug apparent solubility. The potential biopharmaceutical implications of MgSt variability and variation on drug apparent solubility were investigated by choosing compounds of varying physicochemical properties (drug ionization, drug lipophilicity, drug aqueous solubility) and media (compendial and biorelevant) representing the gastric and intestinal conditions. The criticality of certain variables (drug properties, excipient presence, medium characteristics) on the impact of MgSt on drug apparent solubility was examined with the use of multivariate data analysis (partial least squares (PLS)) and the construction of roadmaps.

## MATERIALS AND METHODS

### Materials

APIs: sulfamethoxazole (SMX) and paracetamol (PRC) were obtained from Fisher Scientific (UK). Furosemide (FRS), itraconazole (ITZ) and dipyridamole (DPL) were obtained from VWR (UK). Ibuprofen (IBU), carbamazepine (CBZ) and metformin (MTF) were obtained from Fagron (UK). Excipients: MgSt-BDH was obtained from BDH chemical Ltd. (UK). Ligamed MF-2-V and Ligamed MF-3-V were obtained from Peter Greven (Germany). Chemicals: acetic acid (> 99.7%), hydrochloric acid 36.5–38%, high-performance liquid chromatography (HPLC) grade methanol, HPLC grade acetonitrile, dichloromethane, heptane, pepsin (from porcine) were obtained from Sigma-Aldrich (UK). Maleic acid, sodium chloride, sodium hydroxide, potassium phosphate monobasic, sodium dihydrogen orthophosphate dihydrate, disodium hydrogen orthophosphate dihydrate, potassium dihydrogen orthophosphate, anhydrous sodium sulfate, HPLC grade trifluoroacetic acid, stearic acid > 98% and palmitic acid > 98% were obtained from Fisher Scientific (UK). Boron trifluoride-methanol complex 12% in methanol was obtained from VWR (UK). Sodium taurocholate (Prodotti Chimici Alimentari S.P.A., Italy), egg lecithin-Lipoid EPCS (Lipoid GmbH, Germany), were obtained from the sources specified. Water was ultra-pure (Milli-Q) laboratory grade. Filters: Whatman® 13 mm cellulose nitrate filters 0.45 μm pore size and polytetrafluoroethylene (PTFE) 13 mm filter 0.45 μm pore size were purchased from Fisher Scientific (UK).

### Instrumentation

Fisherbrand waterbath (Fisher Scientific, UK), Sartorius BP 210 D balance (Sartorius Ltd. UK), Buchi R114 Rotavapor (Buchi, Switzerland), SevenCompact S210 pH meter (Mettler Toledo, Switzerland), Vortex-Genie 2 vortex mixer (Scientific Industries Inc., USA), D8 Advance AXS Bruker Powder diffractometer (Bruker Inc., USA), Agilent Technologies 1100 series HPLC system, (quaternary pump (G1311A), autosampler (G1313A), thermostatted column compartment (G1316A), diode array detector (G1329A), Chemstation software (Agilent Technologies, USA) and Chrompack CP-9003 series GC system coupled with a Varian 4400 integrator (Varian Instruments, USA).

### Methods

#### Compounds Selected for Solubility Experiments

The compounds were selected to cover a wide range of properties in terms of ionization (low ionized: *F*_(ion)_ < 50%, highly ionized: *F*_(ion)_ > 50%), lipophilicity (based on the drugs’ partition coefficient (log *P*): − 1.5 < log *P* < 6.5) and aqueous solubility (based on the compound’s BCS (Biopharmaceutical Classification System) classification (high: BCS class I and III; low: BCS class II and IV)) [20]. These properties were selected based on their role on oral drug absorption [21]. The compounds used for the solubility experiments, their physicochemical properties (drug ionization, drug lipophilicity, drug aqueous solubility) and their structure are presented in Table I.

**Table I:**
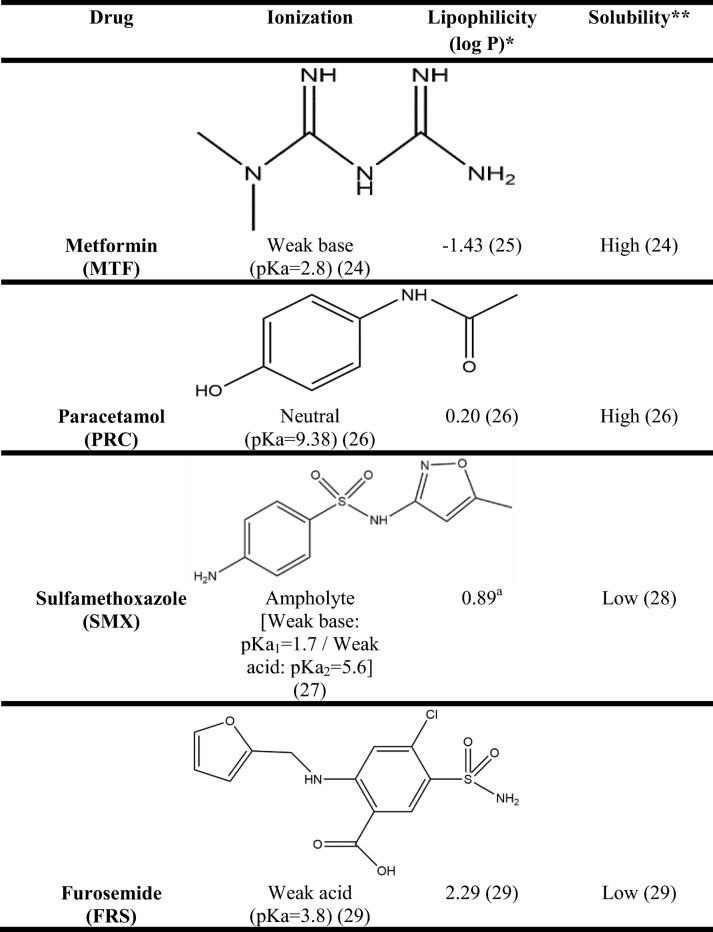

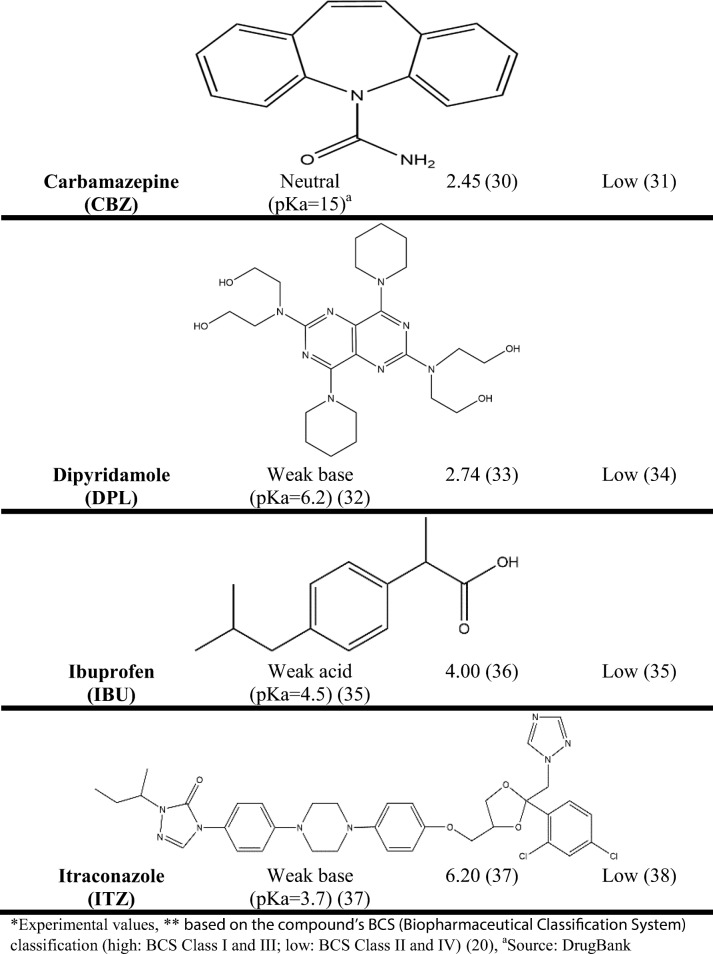
Physicochemical Properties and Structure of the Compounds Used for the Solubility Experiments (ChemDraw Professional 15)

*Experimental values

**Based on the compound’s BCS (Biopharmaceutical Classification System) classification (high: BCS class I and III; low: BCS class II and IV) ( [20])

^*a*^Source: DrugBank

#### Media Prepared for Solubility Experiments

Compendial media (0.1 N HCl pH 1, phosphate buffer pH 6.8) were prepared according to the method described in the European Pharmacopeia [22]. Fasted-State Simulated Gastric Fluid (FaSSGF) and Fasted-State Simulated Intestinal Fluid (FaSSIF-V2) were prepared as described by Jantratid *et al.* [23].

#### Design of Experiments (DoE) Used for Solubility Studies

A full-factorial Design of Experiments (DoE) was performed to determine the number of necessary experiments using StatGraphics Centurion XVII (Statpoint Technologies Inc., USA). As drug solubility will differ according to the composition of the studied media (pH, presence of bile salts), two models for the DoE were constructed to discriminate between the effects of excipients on drug apparent solubility in compendial (model 1) and biorelevant conditions (model 2). The investigated factors were (i) compound (Table I), (ii) excipient brand (MgSt-BDH, Ligamed MF-2-V, Ligamed MF-3-V), (iii) excipient level (low, high) and (iv) medium (gastric, intestinal). The impact of each excipient on drug apparent solubility (expressed as the relative increase or decrease in presence compared with the absence of excipient (Treatment of *in vitro* solubility data)) was set as the response. A total of 96 × 3 experiments was determined for each model. A total of 16 × 3 additional experiments for each model were conducted to determine drug solubility in the corresponding media in the absence of excipient. These experiments were not included in the DoE as drug solubility in excipient absence was measured only for the calculation of relative excipient effects on drug apparent solubility.

#### Characterization of MgSt

##### Determination of Relative Content of Stearic and Palmitic Acid

The relative content and total sum of stearic and palmitic acid for each MgSt brand were determined after esterification of the fatty acids with methanol/boron trifluoride followed by gas chromatography according to the proposed method in the USP monograph of MgSt [39].

##### Powder X-Ray Diffraction

X-ray powder diffraction (XRPD) measurements for each MgSt brand were obtained. Samples were mounted on an acrylic wafer and analysed at *λ* = 1.5418 Å. Samples were measured in reflection geometry in the θ–2θ configuration over the scan range 2 to 40°θ with a 0.12-s exposure per 0.02° increment. The X-rays were generated by a Cu long-fine focus tube operated at 40 kV and 40 mA. Excipient CRS was determined by the intensity of diffractograms (counts) at the 5° 2θ scale.

##### Particle Size Distribution and Specific Surface Area

The PSD of the MgSt brands was measured using laser diffraction in dry dispersion mode at a dispersion pressure of 0.5 bars. The cumulative undersized particle parameters d_10_ (μm), d_50_ (μm) and d_90_ (μm) were calculated. Morphological evaluation of the studied MgSt brands was conducted using scanning electron microscopy (SEM). The SSA of the studied MgSt brands was determined by nitrogen adsorption using the Brunauer-Emmett-Teller (BET) method. Samples of approximately 1 g were dispensed into 3/8” OD sample tubes and prior to analysis, the tubes were outgassed using a VacPrep 061 (Micromeritics, USA) outgassing unit under vacuum at ambient temperature for a minimum of 15 h. Experiments were conducted using a Tristar 3030 instrument (Micromeritics, USA). For each sample tube, specific surface area was calculated from a linear fit of the adsorption isotherm in a relative pressure range of 0.05–0.30 Pa. Three samples tubes were tested for each brand of MgSt and the reported results are the mean values of triplicate determinations [40].

#### Solubility Studies

Drug solubility studies in the absence and presence of excipient were performed in triplicate using the shake-flask method [41]. Drug excess amount and 2% w/w or 5% w/w of each excipient were weighed and placed in centrifuge tubes. For poorly soluble drugs, the amount of excipient was determined considering an average of 500 mg tablet weight [42] which resulted in 9% w/w (10 mg of excipient and 100 mg of drug; low level) and 20% w/w (25 mg of excipient and 100 mg of drug; high level) of excipient in the total volume of the physical mixture. For highly soluble drugs, as higher drug excess amount was used to ensure saturation, the excipient amount was increased in order to keep the same percentage weight per weight (% w/w) of excipient in the total volume of the physical mixture as per the poorly soluble drugs. The physical mixtures were vortexed for 3 min. Five millilitres of each medium was added in the tubes and the samples were placed in a shaking water bath (37°C, 200 strokes per minute (spm)). At 0.5, 4 and 24 h (for PRC, SMX, CBZ, DPL, IBU) and at 24 h (for MTF, FRS, ITZ), 500 μL was sampled and filtered through PTFE filters (or cellulose nitrate filters for the cases of IBU and CBZ). Filter adsorption studies were performed prior in triplicate for each drug. No adsorption issues onto the filters used were observed for the studied drugs. Filtered samples were further diluted (if needed) with the corresponding medium and analysed by HPLC (Supplementary Table I). Analytical HPLC procedures for drug quantification in the samples were modifications of already published methods. Drug quantification was made based on calibration curves. Standards were formulated from concentrated stock solutions consisting of drug dissolved in MeOH. As changes in the pH of solutions by the presence of the dissolved drug [34] or MgSt [43, 44] may affect drug solubility, the pH of samples after the completion of each experiment was measured. Drug solubility was calculated based on the sample drug concentration measured. Solubility values measured experimentally for neutral drugs, for weak acids in acidic media and for weak bases in basic media determined the intrinsic solubility values. Solubility values measured experimentally in basic media (for weak acids) and acidic media (for weak bases) determined drug solubility of the ionized molecules. As experimental points over a period of time were not available for the whole set of drugs to ensure that equilibrium solubility has been reached in 24 h for all the studied compounds, the drug solubility measured was considered as the apparent drug solubility (kinetic solubility).

#### Treatment of *In Vitro* Solubility Data

The relative effect (RE) of each excipient on drug apparent solubility was calculated based on Eq. (1):

1 $$\mathrm{RE}=\frac{\left(S- Sr\right)}{Sr}\ x\ 100$$

where *S* and *Sr* denote drug solubility in presence and absence (reference solubility) of excipient at 0.5, 4 and 24 h. REs of excipients on drug apparent solubility > 25% or < − 20% were considered as a significant change in drug solubility to assess excipient criticality (this range was selected as a similar range is set in order to assess differences in drug exposure after oral administration, *i.e.* in bioequivalence studies) [45].

Box plots depicting the impact of excipients on drug solubility at 24 h for all the studied compounds in presence of all the studied MgSt brands or as a function of time (0.5, 4 and 24 h) for PRC, CBZ, SMX, IBU and DPL in presence of MgSt-BDH and Ligamed MF-2-V were constructed using Spotfire 7.10.1 (TIBCO Software Inc., USA). The classification gradient maps portraying the impact of excipients on drug solubility at 24 h as a function of drug aqueous solubility were generated using SigmaPlot 13.0 (Systat Software Inc., USA). For the construction of 3D mesh plots depicting the impact of excipients (Ligamed MF-2-V, Ligamed MF-3-V) on drug solubility at 24 h as a function of drug ionization and drug lipophilicity, the REs on drug solubility were smoothed via the locally weighed scatterplot smoothing (LOWESS) regression to allow better visualization using SigmaPlot 13.0 (Systat Software Inc., USA).

In cases where drug intrinsic solubility was not determined experimentally (SMX and DPL in compendial and biorelevant media), the theoretical intrinsic solubility was calculated using the solubility-pH equations (Eqs. (2)–(5)) [46]:

2 $$\log S=\log {S}_{\mathrm{o}}+\log \left({10}^{-\mathrm{pKa}+\mathrm{pH}}+1\right)\ \mathrm{for}\ \mathrm{weak}\ \mathrm{acids}$$

3 $$\log S=\log {S}_{\mathrm{o}}+\log \left({10}^{\mathrm{pKa}-\mathrm{pH}}+1\right)\ \mathrm{for}\ \mathrm{weak}\ \mathrm{bases}$$

4 $$\log S=\log {S}_{\mathrm{o}}+\log \left({10}^{+\mathrm{p}{\mathrm{Ka}}_2+{\mathrm{pKa}}_1-2\mathrm{pH}}+{10}^{{\mathrm{pKa}}_2-\mathrm{pH}}+1\right)\ \mathrm{for}\ \mathrm{diprotic}\ \mathrm{bases}$$

5 $$\log S=\log {S}_{\mathrm{o}}+\log \left({10}^{+{\mathrm{pKa}}_1-\mathrm{pH}}+{10}^{-{\mathrm{pKa}}_2+\mathrm{pH}}+1\right)\ \mathrm{for}\ \mathrm{ampholytes}$$

where *S* and *S*_o_ indicate the thermodynamic solubility at the given pH and the intrinsic solubility, respectively. These equations provide a simplified view for the determination of drug solubility as deviations from these models (in cases of drug aggregation or drug solubilization in the biorelevant media) can be anticipated [46]. The final pH and experimental solubility values of the ionized drug in basic (for weak acids) or acidic media (for weak bases) were used for the calculation of the theoretical intrinsic solubility. Theoretical pH-solubility profiles in the physiological pH range were constructed to assess if changes in the pH of the medium could justify differences in drug solubility by excipient presence. The final pH and intrinsic solubility values (experimental or theoretical) were used for the construction of the theoretical pH-solubility profiles in the physiological pH range based on Eqs. (2)–(5).

#### Multivariate Analysis of *In Vitro* Solubility Data

Excipient REs on drug apparent solubility were correlated to drug physicochemical properties (drug ionization, drug lipophilicity, drug aqueous solubility), excipient critical material attributes (CRS, PSD, level) and medium characteristics (gastric, intestinal) by partial least squares (PLS) regression using the XLSTAT software (Microsoft, USA). Two models for the REs of excipients on drug apparent solubility in compendial media (model 1) and biorelevant media (model 2) were constructed. The evaluated variables for both models were categorized according to their type as categorical (expressing a category or type) and numerical (measurements with numerical meaning). Categorical variables included were (i) drug solubility (low, high), (ii) excipient level (low, high) and (iii) medium (gastric, intestinal) while numerical parameters included were (i) theoretical % of drug ionized (*F*_ion_; calculated based on the Henderson-Hasselbalch equation at the pH of each medium), (ii) drug lipophilicity (log *P*), (iii) excipient CRS and (iv) excipient PSD. Excipient REs on drug solubility at 24 h were used as the response. The selected interaction terms included each excipient property combined with each drug physicochemical property (drug ionization, drug lipophilicity, drug aqueous solubility) and medium characteristics (gastric, intestinal). Observation diagnostics were performed prior to model analysis to identify outliers in the data set. The distance of each observation to the model in the Y-plane (DmodY) tool based on PLS residuals was used. Plots of standardized DmodY *vs* each observation were generated and any observation exceeding the maximum tolerance volume in Y (D_crit(Y)_) was considered an outlier [47, 48]. Exclusion of outliers was based on (i) deviating cases (positive REs) in solubility caused by a shift in the pH of the solution and (ii) observations resulting in high variability (coefficient of variation (CV%) > 20%) within the triplicate samples (one value from the triplicate could be excluded as the outlier analysis could detect these values). PLS models generated with and without outlier exclusion (data not shown) confirmed that outlier exclusion did not alter the interpretation of results but only enhanced the predictive ability of the regression model. The generated models were assessed in terms of goodness of fit (*R*^2^) and goodness of prediction (*Q*^2^). High values of *R*^2^ and *Q*^2^ with a difference not greater than 0.2–0.3 were indications of successful models [49]. The number of PLS components (lines on the X-space which best approximate and correlate with the Y-vector) was based on the minimum predictive residual sum of squares (PRESS) [49]. From the available components, the one at which *Q*^2^ reached its maximum value was selected [47]. Standardized coefficients were used to show the direction (positive or negative) and extent of each variable on the response. The significance of the variables was assessed by the variable influence on projection (VIP) value. VIP values > 0.8 were considered moderately influential in the model while VIP values > 1 were considered the most influential in the model [49]. A 95% confidence interval was used.

#### Roadmap Design

A roadmap design for the identification of potential risks of MgSt variability on drug apparent solubility was constructed combining the impact of excipients on drug solubility at 24 h from the solubility studies to excipient (CRS, PSD) and drug (drug ionization, drug lipophilicity, drug aqueous solubility) characteristics. Drugs were categorized according to drug aqueous solubility and drug lipophilicity (Table I) and drug ionization (low ionized: *F*_(ion)_ < 50%, highly ionized: *F*_(ion)_ > 50%). The risk assessment of the impact of excipients on drug apparent solubility was evaluated by setting reference range criteria of − 20 to 25% [45] on the REs of excipients on drug solubility. REs of excipients on drug solubility outside these values (REs < − 20% or REs > 25%) were considered to be potentially significant for oral drug performance.

## RESULTS AND DISCUSSION

### Characterization of MgSt

#### Determination of Relative Content of Stearic and Palmitic Acid

The relative content and total sum of stearic and palmitic acid for each MgSt brand are presented in Fig. 1b. The relative content varied between 70%:30% of stearic:palmitic acid for MgSt-BDH and Ligamed MF-3-V and 65%:35% of stearic:palmitic acid for Ligamed MF-2-V. The total sum of the fatty acids of the studied brands was > 99.99%, within the acceptance criteria of the USP monograph of MgSt [39]. As changes in the lubrication efficiency of MgSt have been reported only for differences between the stearic and palmitic relative contents higher than 20% [16], the potential changes in drug solubility by the studied brands in this study are unlikely to be attributed to the small differences in the relative fatty acid content of the studied brands. Evaluation of MgSt brands with significantly different stearic and palmitic acid content could be used in future studies to assess the biopharmaceutical implications of this excipient property.

#### Powder X-Ray Diffraction

The XRPD patterns of MgSt-BDH, Ligamed MF-2-V and Ligamed MF-3-V are shown in Fig. 1c. The patterns are in accordance with previously reported diffractograms [15]. The intensity and sharpness of the peaks at the 3.3° 2θ, 5.5° 2θ and 23° 2θ scale revealed higher crystalline portions for Ligamed MF-2-V and Ligamed MF-3-V compared with MgSt-BDH.

#### Particle Size Distribution

The PSD and SSA analysis of the studied MgSt brands are shown in Fig. 1d. Ligamed MF-3-V comprised of smaller particles compared with MgSt-BDH and Ligamed MF-2-V. SEM micrographs confirmed these findings (Fig. 1e) as large agglomerates were observed for MgSt-BDH and Ligamed MF-2-V. The SSA was higher for Ligamed MF-3-V compared with MgSt-BDH and Ligamed MF-2-V, due to its lower PSD. Differences in crystallinity between MgSt-BDH and Ligamed MF-2-V (expressed by the differences in the intensity and sharpness of diffractogram peaks, Fig. 1c) could justify the lower SSA of MgSt-BDH compared with Ligamed MF-2-V, despite its lower PSD [50].

### Solubility Studies

#### Impact of the Studied MgSt Brands on Drug Apparent Solubility

**Fig. 2:**
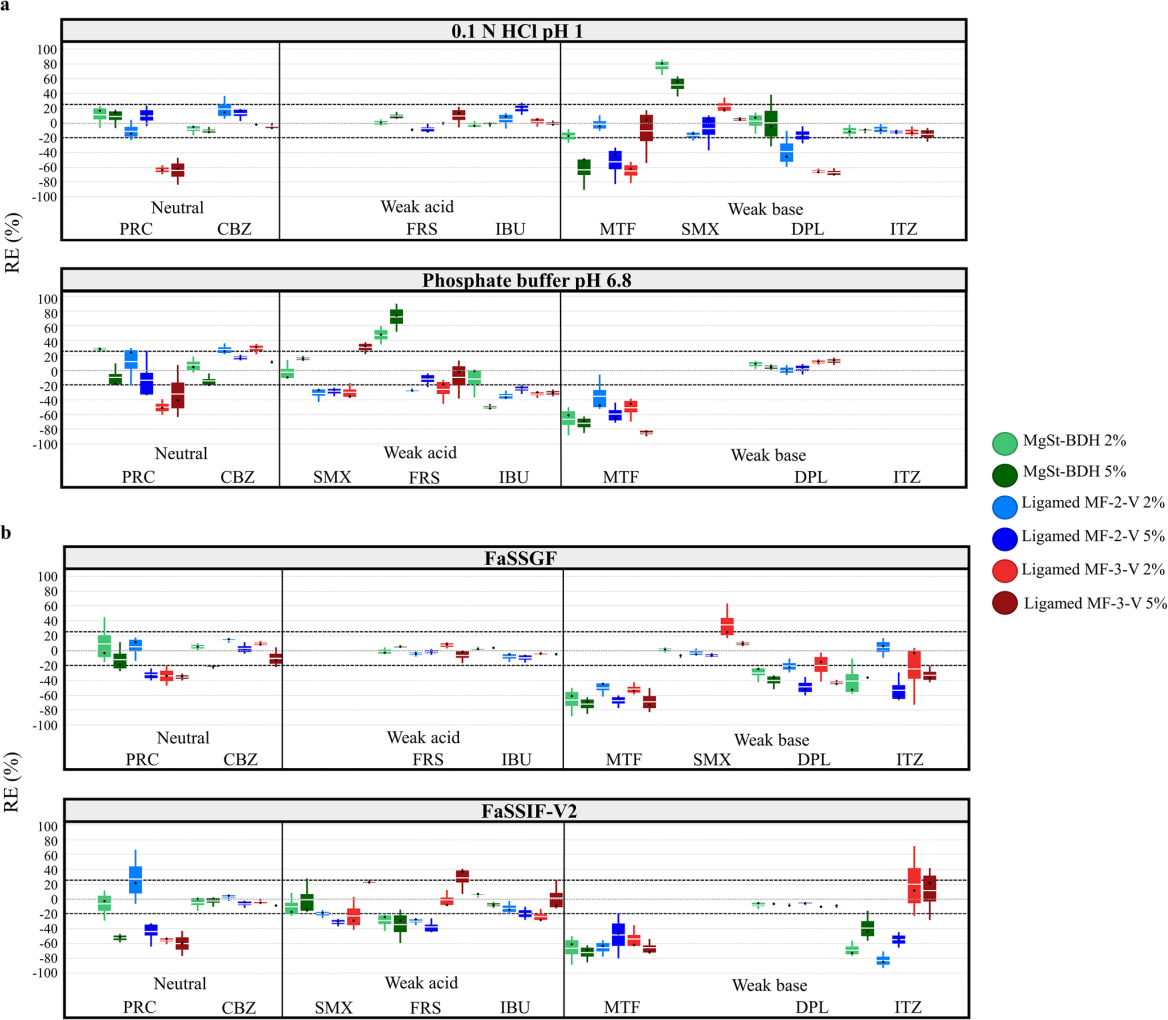
Box plots of the relative effects (%) of the MgSt brands on drug solubility at 24 h in **a** compendial and **b** biorelevant media. The excipient brands are shown as (i) MgSt-BDH (green colour), (ii) Ligamed MF-2-V (blue colour) and (iii) Ligamed MF-3-V (red colour). Light and dark colours correspond to low and high excipient level, respectively (mean (white line), median (black diamond), *n* = 3)

The reference drug solubility values at 24 h in compendial and biorelevant media are summarized in Table II. The reference solubility of neutral drugs (PRC, CBZ) was not pH-dependent, as expected. Higher solubility values were observed in biorelevant compared with compendial media for neutral drugs, as the presence of bile salts improves drug solubilization [51]. Weak acids (SMX in basic media, FRS, IBU) and weak bases (SMX in acidic media, DPL, ITZ) showed a pH-dependent solubility in the physiological pH range due to their ionization pattern. The solubility of MTF (weak base) was not pH-dependent, as drug was fully ionized in the studied conditions [24]. Due the presence of two pKa constants (Table I), SMX is ionized in acidic (80% and 50% of drug ionized in 0.1 N HCl pH 1 and FaSSGF pH = 1.6, respectively) and basic media (92% and 86% in phosphate buffer pH 6.8 and FaSSIF-V2 pH = 6.5, respectively). The reference solubility values of SMX in acidic media were lower compared with their corresponding basic media due to the higher percentage of ionized drug in basic compared with acidic conditions. In media where weak acids or weak bases were unionized, drug solubility was higher in biorelevant compared with compendial media due to the presence of solubilizing components [51]. In media, where weak acids and weak bases (except the case of MTF) are ionized, drug solubility values were higher in the compendial compared with the biorelevant conditions, despite the absence of solubilizing components. These changes in drug solubility can be explained by the differences in the pH between the studied sets of media which result in a higher percentage of drug ionized in compendial compared with biorelevant conditions and therefore higher reference drug solubility [52]. The effects of the MgSt brands on the solubility of the studied compounds at 24 h in compendial and biorelevant media are presented in Fig. 2.

**Table II:** Reference Solubility Values (μg/mL) of the Studied Drugs at 24 h in Compendial and Biorelevant Media (mean ± SD, *n* = 3)

Drug	Compendial media		Biorelevant media	
	0.1 N HCl pH 1	Phosphate buffer pH 6.8	FaSSGF	FaSSIF-V2
MTF	3.1 × 10^5^ (± 0.3 × 10^5^)	3.1 × 10^5^ (± 0.2 × 10^5^)	3.4 × 10^5^ (± 0.8 × 10^5^)	4.3 × 10^5^ (± 0.4 × 10^5^)
PRC	1.6 × 10^4^ (± 0.1 × 10^4^)	1.5 × 10^4^ (± 0.1 × 10^4^)	1.7 × 10^4^ (± 0.2 × 10^4^)	1.7 × 10^4^ (± 0.1 × 10^4^)
SMX	1.6 × 10^3^ (± 0.1 × 10^3^)	3.7 × 10^3^ (± 0.1 × 10^3^)	862 (± 21)	1.3 × 10^3^ (± 0.1 × 10^3^)
FRS	14 (± 2)	3.4 × 10^3^ (± 1.4 × 10^2^)	15 (± 1)	1.6 × 10^3^ (± 3.0 × 10^2^)
CBZ	265 (± 6)	227 (± 9)	368 (± 1)	280 (± 7)
DPL	1.3 × 10^4^ (± 9.1 × 10^2^)	5 (± 1)	8.6 × 10^3^ (± 2.0 × 10^2^)	13 (± 1)
IBU	43 (± 3)	5.5 × 10^3^ (± 6.7 × 10^2^)	44 (± 5)	1.5 × 10^3^ (± 5.8)
ITZ	11 (± 1)	-*	1.2 (± 0.2)	0.05 (± 0.01)

*Below limit of detection of the analytical method. *MTF*, metformin; *PRC*, paracetamol; SMX, sulfamethoxazole; *FRS*, furosemide; *CBZ*, carbamazepine; *DPL*, dipyridamole; *IBU*, ibuprofen; *ITZ*, itraconazole

##### Neutral Drugs

**Fig. 3:**
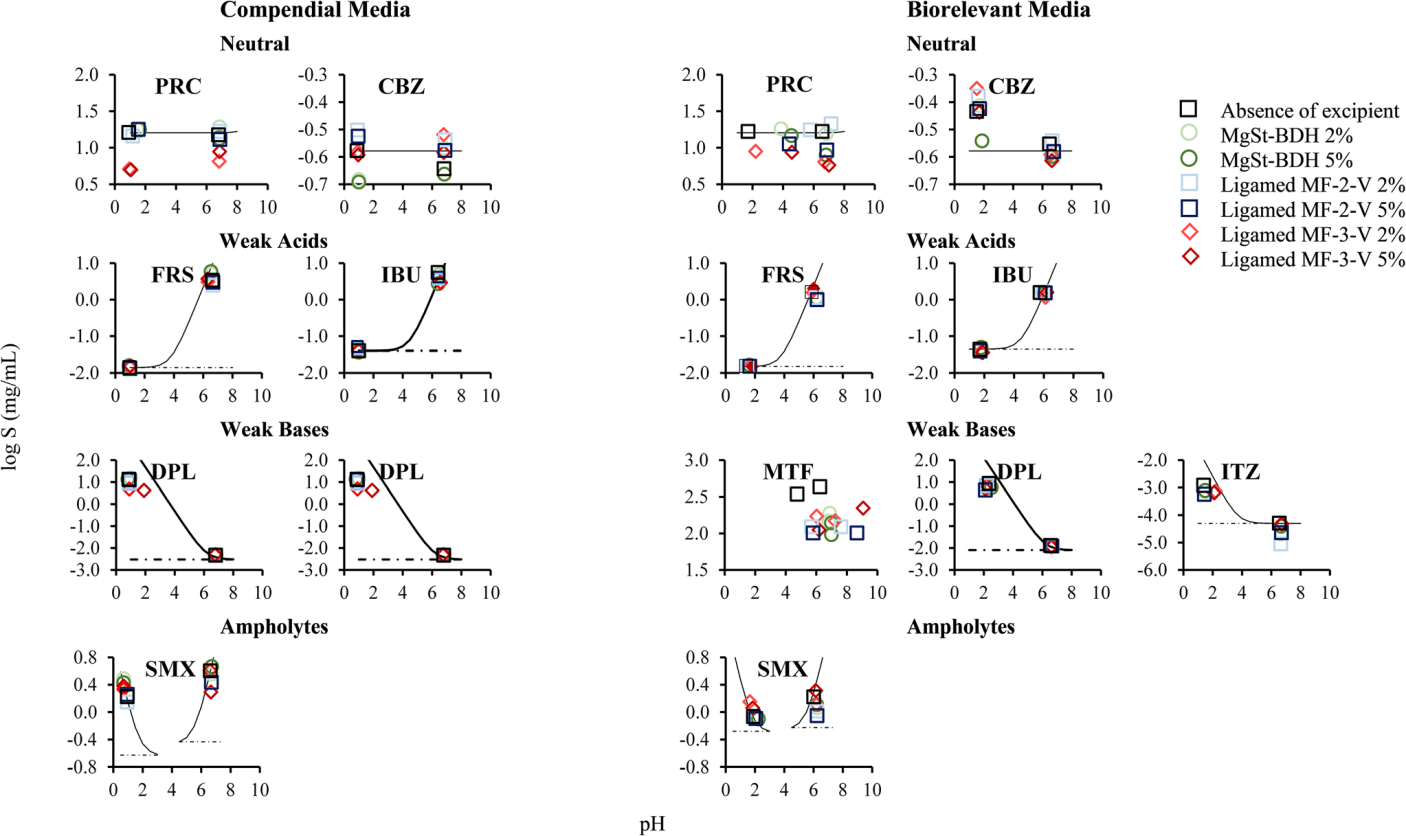
Theoretical pH solubility profiles of the studied drugs in compendial and biorelevant media and experimental solubility values of the studied drugs in absence (black colour) and presence of excipients ((i) MgSt-BDH (green colour), (ii) Ligamed MF-2-V (blue colour), (iii) Ligamed MF-3-V (red colour)). Light and dark colours correspond to low and high excipient level. Dashed lines indicate drug intrinsic solubility

**Fig. 4:**
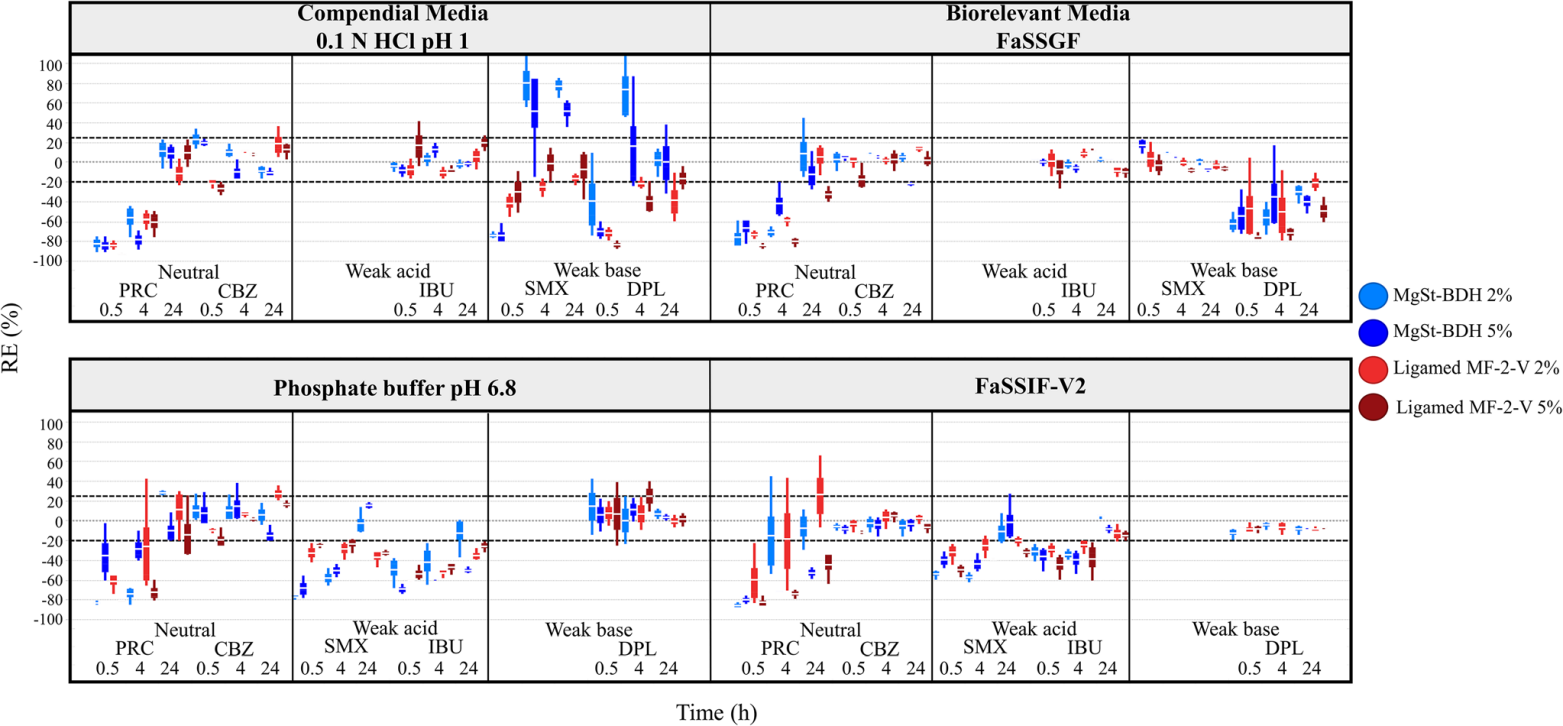
Box plots of relative effects (%) on the solubility of PRC, CBZ, SMX, IBU, DPL in compendial and biorelevant media as a function of time (h) in presence of (i) MgSt-BDH (blue colour) and (ii) Ligamed MF-2-V (red colour). Light and dark colours correspond to low and high excipient level, respectively (mean, *n* = 3)

Significant decrease in drug solubility at 24 h (− 65% < REs < − 32%) by the studied MgSt brands was observed for the case of PRC. For PRC, an increase in the pH of the media was observed in presence of MgSt in 0.1 N HCl pH 1 (0.1–0.6 pH units) and in biorelevant media (0.3–5 pH units) due to the basic nature of the excipient [43, 44]; however, changes in the pH of the media cannot justify the differences in solubility in MgSt presence (Fig. 3). The reduction in PRC solubility at 24 h by MgSt can be explained by the decreased drug-medium contact in excipient presence [53]. The hydrophobic nature and slow dissolution of MgSt from the powder surface [7, 8] which further limit drug dissolution and/or drug solubilization could also contribute to the decrease in drug apparent solubility. The effects of MgSt-BDH and Ligamed MF-2-V on drug solubility as a function of time for one highly soluble (PRC) and 4 poorly soluble drugs (CBZ, SMX, IBU, DPL) in compendial and biorelevant media are presented in Fig. 4. The presence of MgSt resulted in a pronounced decrease in PRC solubility (− 90% < REs < − 60%) at early time points (0.5 and 4 h) confirming that the reduction in drug solubility by MgSt presence relates to the lipophilic nature of the excipient. Significant increase in drug solubility at 24 h was observed in phosphate buffer pH 6.8 for PRC (2% MgSt-BDH: RE = 28%) and for CBZ (2% of Ligamed MF-2-V: RE = 29%, Ligamed MF-3-V: RE = 27%) and in FaSSIF-V2 for PRC (2% of Ligamed MF-2-V: RE = 27%) (Fig. 2). The observed slight differences in the pH of the media (0–0.2 pH units) due to the presence of MgSt [43, 44] are not expected to affect the apparent solubility of neutral drugs (Fig. 3). As MgSt particles disperse in basic conditions [19], the observed increase in drug solubility at 24 h could be attributed to improved drug powder dispersion which facilitates drug solubilization.

##### Weak Acids

Cases of a significant reduction in the solubility of weak acids at 24 h by MgSt presence were observed only in basic conditions (SMX: − 30% < REs < − 20%, FRS: − 40% < REs < − 25%, IBU: − 50% < REs < − 25%) (Fig. 2). For cases where MgSt presence significantly decreased drug solubility, small changes in the pH of the acidic media occurred in absence or presence of excipient (± 0.1 pH units) while the pH of the basic media decreased in absence or presence of excipient (0.2–0.7 pH units), potentially due to the dissociation of weak acids. The construction of the theoretical pH-solubility profiles (Fig. 3) revealed that in cases where MgSt significantly reduced drug apparent solubility, the changes in the pH of media in excipient presence cannot justify the changes in drug solubility at 24 h, as the experimental solubility values in excipient presence do not correspond to the theoretical solubility values (expected according to the changes in the pH of the media). The reduced drug dissolution and/or drug solubilization by the lipophilic MgSt [7, 8, 53] can explain the observed reduction in drug apparent solubility by MgSt, as in the case of neutral drugs. The impact of MgSt-BDH and Ligamed MF-2-V on the solubility of two weak acids (SMX in basic media and IBU) through time (Fig. 4) showed that the reduction in drug solubility by MgSt was higher at early (− 80% < REs < − 20%) compared with late time points (24 h), even in several cases where MgSt did not significantly alter the 24-h drug solubility. This observation confirms that the reduction in drug apparent solubility is a result of reduced powder wetting in presence of MgSt. Significant increase in weak acidic drug solubility at 24 h was observed in basic conditions for FRS (MgSt-BDH: REs of 47% and 71% for the low and high level, respectively) and SMX (5% Ligamed MF-2-V: RE = 30%) in phosphate buffer pH 6.8 and for FRS (5% Ligamed MF-3-V: RE = 28%) in FaSSIF-V2. For the above cases, the reduction in the pH of the medium was lower in the presence (approximately 0.3 pH units) compared with excipient absence (0.4–0.6 pH units) potentially due to the basic nature of MgSt [43, 44] and explains the differences in the drug solubility at 24 h between excipient absence and presence (Fig. 3).

##### Weak Bases

Significant reduction in the solubility of weak bases at 24 h by MgSt presence was observed for the majority of cases (MTF: − 84% < REs < − 35%, DPL: − 67% < REs < − 20%, ITZ: − 50% < REs < − 20%) (Fig. 2). The impact of pH on the solubility of the aforementioned drugs cannot be evaluated, as the theoretical pH-solubility profiles did not follow the exponential increase in drug solubility with increasing pH due to *in situ* salt formation between the drugs and counterions of the medium [54] (Fig. 3). The hydrophobic nature of MgSt [7, 8, 53] leading to slow drug dissolution and/or drug solubilization could justify the decrease in drug apparent solubility. The impact of excipients (MgSt-BDH, Ligamed MF-2-V) on the solubility of two weak bases (SMX in acidic media and DPL) through time (Fig. 4) showed a pronounced reduction in drug solubility at early time points mainly in acidic media confirming the negative impact of MgSt on drug dissolution/solubilization (only in presence of BDH an increase was observed in drug solubility at 4 h for SMX and DPL and at 24 h for SMX in 0.1 N HCl pH 1). Significant increase in drug solubility at 24 h in acidic media was observed for SMX in 0.1 N HCl pH 1 (MgSt-BDH: REs of 77% and 52% for the low and high level, respectively) and in FaSSGF (2% Ligamed MF-3-V: RE = 34%) (Fig. 2). In the aforementioned cases, the pH of the medium in presence was lower (0.1 N HCl pH 1: pH = 0.7 in presence of MgSt-BDH, FaSSGF: pH = 1.64 in presence of Ligamed MF-3-V) compared with excipient absence (0.1 N HCl pH 1: pH = 0.94, FaSSGF: pH = 1.89) (Fig. 3). The increase in drug apparent solubility is therefore attributed to the change in the pH of the media as experimental and theoretical drug solubilities at 24 h in excipient presence are identical (further investigations of the interplay between drugs or excipients and the pH of the medium are needed as MgSt is not expected to reduce the pH of the medium).

The solubility data showed increased variability in the cases where MgSt presence significantly affected drug solubility (PRC, MTF: CV% > 40%, highly ionized drugs: 20% < CV% < 40%). As working with physical mixtures may yield high standard deviations due to the heterogeneous dispersion of the constituents [55, 56], the increased variability can be attributed to the heterogeneous saturation of powder surface with excipient particles.

#### Impact of Excipients on Drug Apparent Solubility Based on Drug Physicochemical Properties

**Fig. 5:**
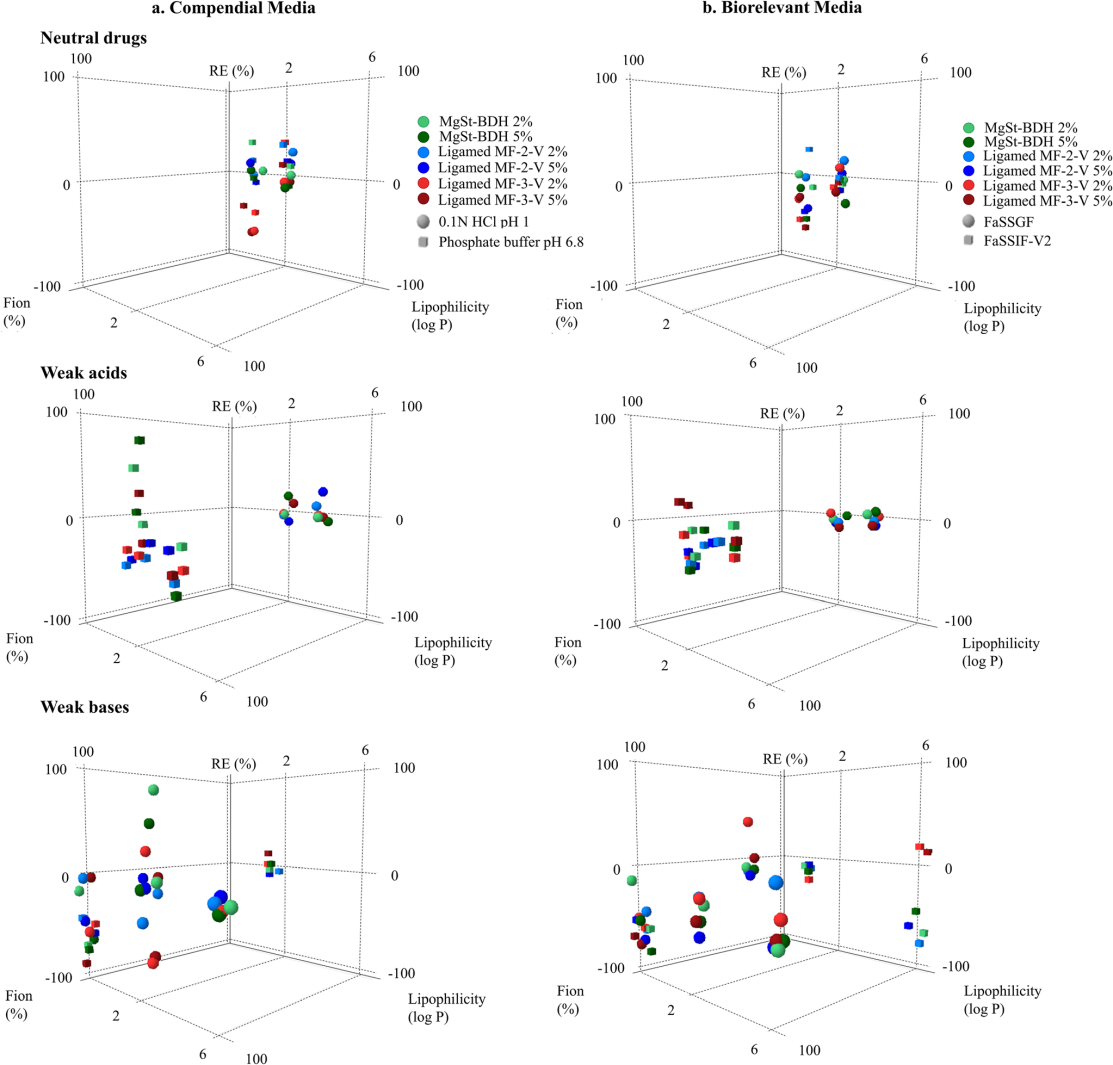
Relative effects (%) of MgSt brands on drug solubility at 24 h as a function of drug ionization (%) and drug lipophilicity (log *P*) in **a** compendial and **b** biorelevant media. The excipients brands are shown as (i) MgSt-BDH (green colour), (ii) Ligamed MF-2-V (blue colour), (iii) Ligamed MF-3-V (red colour). Light and dark colours correspond to low and high excipient levels, respectively. Media representing gastric and intestinal conditions are presented as (i) acidic media (circles) and (ii) basic media (squares)

**Fig. 6:**
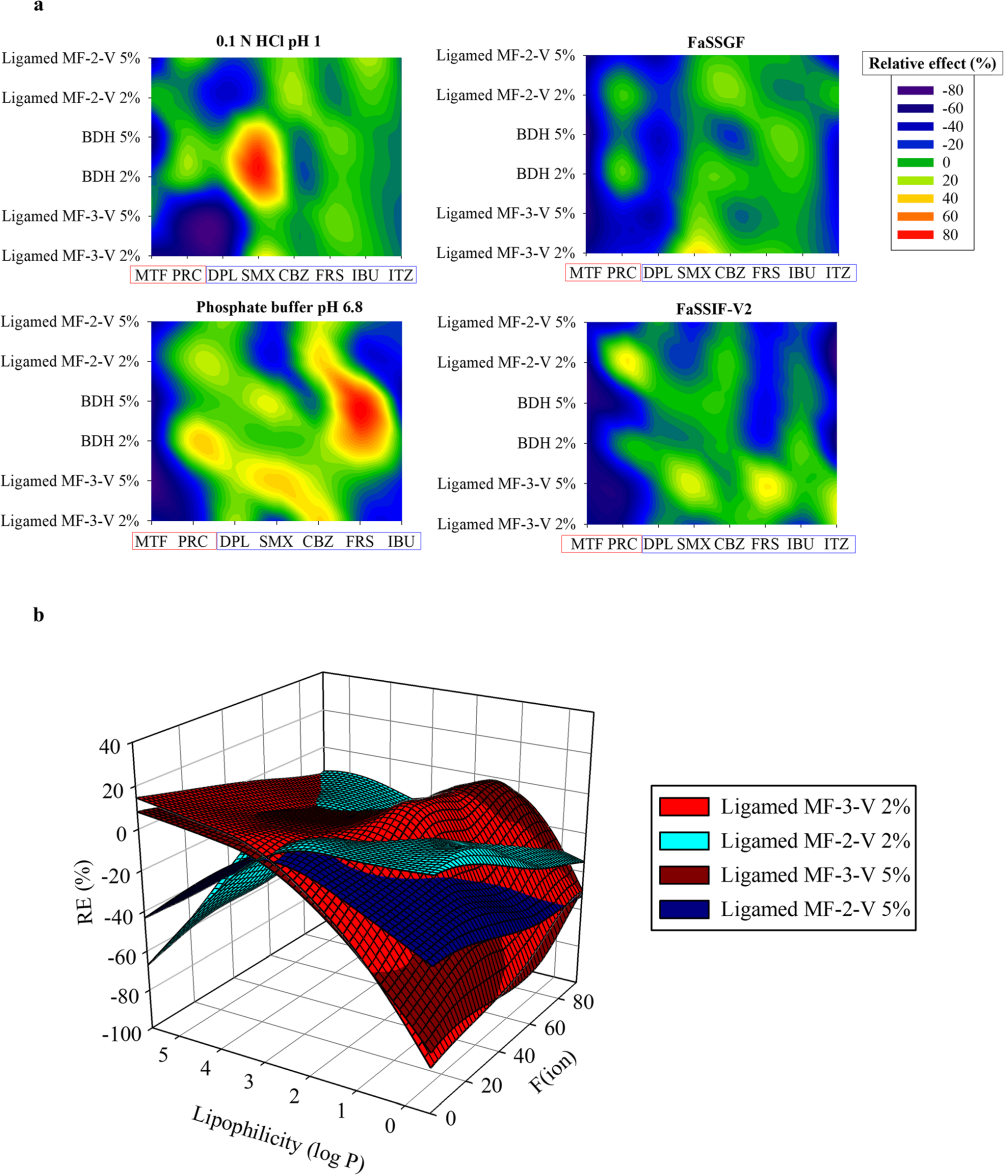
**a** Classification gradient maps of the relative excipient effects of the MgSt brands on the solubility of highly and poorly soluble compounds at 24 h. Y-axes are set in an increasing excipient particle size and excipient level order. The x-axes are set in a decreasing drug aqueous solubility order (red colours for highly soluble and blue colours for poorly soluble drugs). **b** Relative effects (%) of (i) Ligamed MF-2-V (blue colour) and (ii) Ligamed MF-3-V (red colour) on drug solubility at 24 h as a function of drug ionization (%) and lipophilicity (log *P*). Light and dark colours correspond to low and high excipient level, respectively

The effects of MgSt on the solubility of neutral drugs, weak acids and weak bases at 24 h as a function of drug ionization and drug lipophilicity in compendial and biorelevant media are presented in Fig. 5. As both neutral drugs are unionized under the studied media, the higher decrease in the apparent solubility of PRC compared with CBZ by MgSt is likely attributed to the differences in the lipophilicity of these two compounds (Table I). For weak acids and weak bases, the decrease in drug apparent solubility is pronounced in media where drugs are highly ionized (excluding the cases of increased drug solubility attributed to the change of the pH of the medium). A clear trend between the impact of excipients on the 24 h solubility of weak acids and bases and drug lipophilicity cannot be observed. The decrease in drug apparent solubility by MgSt presence was more pronounced with increasing level of drug ionization and/or decreasing drug lipophilicity (Fig. 5). As in these cases a higher number of drug molecules can dissolve fast in the surrounding medium, the powder surface can saturate with the lipophilic MgSt particles and results in decreased drug apparent solubility [19]. The classification gradient map (Fig. 6a) depicting the effects of the MgSt brands on drug solubility at 24 h as a function of decreasing drug aqueous solubility in compendial and biorelevant media confirms this finding. The presence of MgSt decreased the 24-h solubility of drugs with high aqueous solubility. For the majority of drugs with low aqueous solubility, drug solubility was not affected by excipient presence, except for the cases of highly ionized drugs.

#### Impact of Excipients on Drug Apparent Solubility Based on Excipient Properties

##### Impact of MgSt Crystallinity on Drug Apparent Solubility

The effects of the studied MgSt brands on drug solubility depended on excipient properties. For the majority of cases, a significant reduction in drug apparent solubility was observed mainly in presence of high crystalline brands (Ligamed MF-2-V, Ligamed MF-3-V) compared with the low crystalline brand (MgSt-BDH) (Fig. 2). In cases where all the studied brands decreased drug solubility at 24 h, the reduction in drug solubility was more pronounced by the high crystalline (− 85% < REs < − 20%) compared with the low crystalline brand (− 70% < REs < − 20%). Investigation of the impact of the low (MgSt-BDH) and a high crystalline (Ligamed MF-2-V) brand on drug solubility through time (Fig. 4) showed that the crystalline brand resulted in the same or higher reduction in drug solubility at early time points (0.5 and 4 h) compared with the less crystalline brand (except from the case of SMX in 0.1 N HCl pH 1 and phosphate buffer pH 6.8). The better lubrication efficiency of crystalline brands around particles during mixing could explain the more pronounced delay on drug dissolution [18].

##### Impact of MgSt PSD and Level on Drug Apparent Solubility

As the impact of PSD on the lubrication efficiency of MgSt will have greater magnitude for crystalline brands [18], the effects of increasing MgSt particle size on drug apparent solubility were investigated for the two crystalline MgSt brands (Ligamed MF-2-V and Ligamed MF-3-V). The effects of Ligamed MF-2-V and Ligamed MF-3-V on drug solubility at 24 h as a function of drug ionization and drug lipophilicity are presented in Fig. 6b.

Differences in the REs of excipients on drug apparent solubility within the two brands are observed in the low drug ionization range. Presence of the low particle size brand (Ligamed MF-3-V) reduced the solubility of drugs with log *P* < 1 in a higher extent (REs < − 60%) compared with the high particle size brand (Ligamed MF-2-V) (REs < − 40%) at 24 h. The higher reduction in drug apparent solubility in presence of Ligamed MF-3-V compared with Ligamed MF-2-V can be explained by the creation of a stronger and uniform coating around drug particles by MgSt of low particle size [11]. The opposite is observed when increasing drug lipophilicity (log *P* > 4, ITZ). Based on our complete data set, the presence of MgSt should not affect the solubility of poorly soluble unionized drugs (Fig. 5); therefore, further investigations are needed to explain why low particle size brands affected the solubility of ITZ in media where the drug was unionized. Increasing excipient level results in a pronounced reduction in drug apparent solubility only for Ligamed MF-2-V. As high particle size brands (Ligamed MF-2-V) are not able to form a strong layer around particles compared with low particle size brands (Ligamed MF-3-V), increasing their amount could lead to significantly higher drug particle coverage [57].

### Multivariate Data Analysis of Solubility Data

**Fig. 7:**
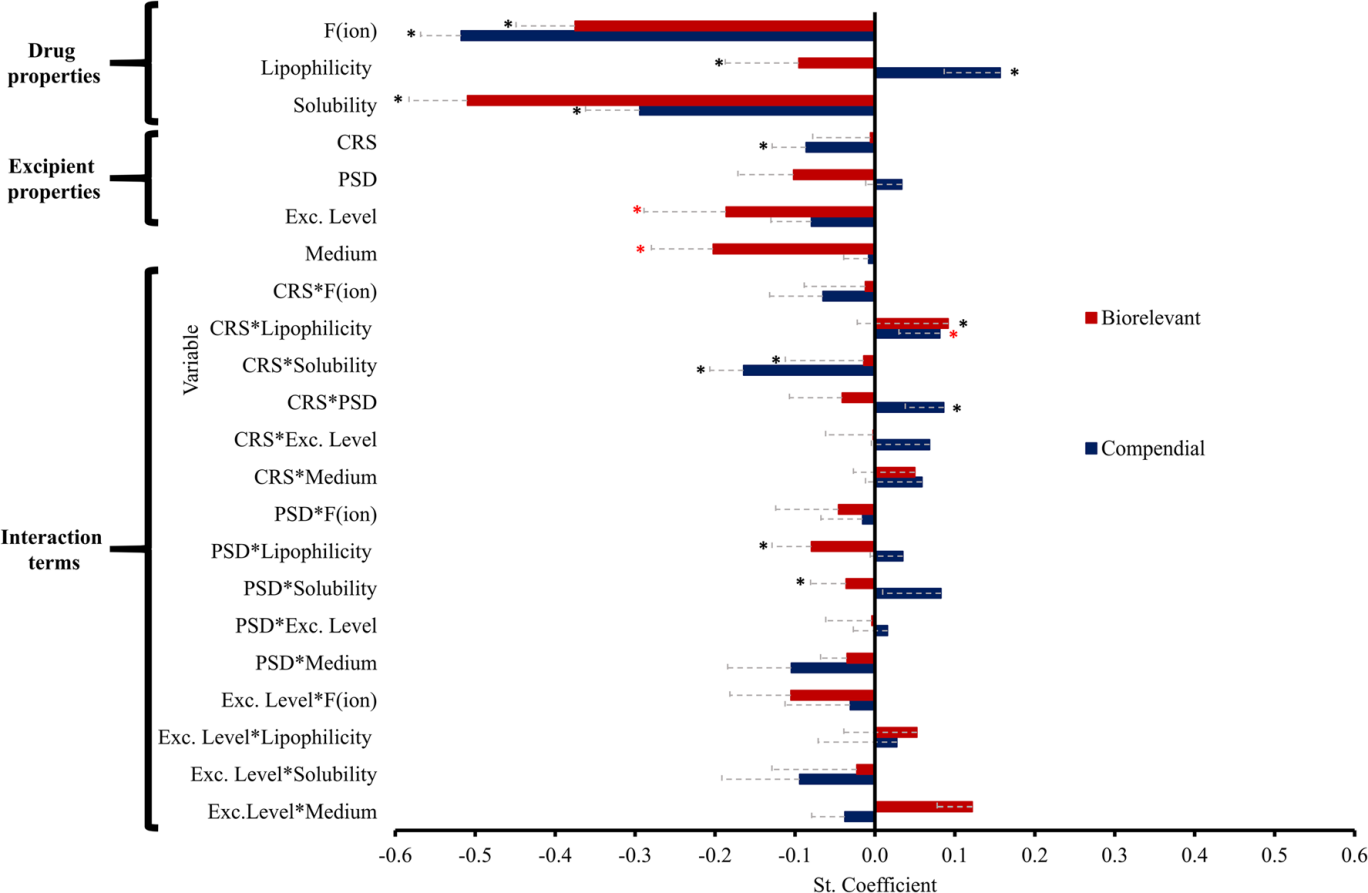
Standardized coefficients of the studied variables (and interaction terms) in compendial (blue colour) and biorelevant (red colour) media. * denotes coefficients of VIP > 1,  denotes coefficients of 0.8 < VIP < 1 (mean ± SE)

The standardized coefficients of the studied variables and their interactions in compendial and biorelevant media are presented in Fig. 7. The two models showed an average predictive power and fit (compendial media: 2 principal components, *Q*^2^ = 0.5 and *R*^2^ = 0.6, biorelevant media: 2 principal components, *Q*^2^ = 0.4 and *R*^2^ = 0.5).

The statistical analysis reveals that the impact of MgSt on drug apparent solubility depends on drug physicochemical properties. Drug ionization (compendial media: negative effect, VIP = 2.7, biorelevant media: negative effect. VIP = 2.0) and drug aqueous solubility (compendial media: negative effect, VIP = 2.0, biorelevant media: negative effect, VIP = 2.9) were influential variables in both sets of media. These variables reveal that the slow dissolution of MgSt from powder surface [53] becomes the rate limiting step for the dissolution of highly ionized and/or highly soluble. Drug lipophilicity was a significant variable in both models with a different extent in compendial (positive effect, VIP = 1.7) and biorelevant media (negative effect, VIP = 1.4), indicating that the decrease in the solubility of lipophilic drugs at 24 h by MgSt presence will be pronounced in biorelevant conditions. The pronounced decrease in the 24-h solubility of lipophilic drugs by MgSt presence in biorelevant media can be explained by the improved solubilization of lipophilic molecules due to the presence of bile salts [51, 58] which results in the presence of more MgSt particles on the surface of the powder. The impact of MgSt on drug apparent solubility relates also to excipient properties according to the sets of media used. Excipient CRS was a significant variable in the compendial model (negative effect, VIP = 1.1) and reveals that the improved lubrication efficiency of crystalline MgSt hinders powder wettability [17, 59]. Excipient CRS was not significant in biorelevant media, as potentially lubricated particles can be solubilized by the presence of bile salts [51, 58]. Excipient level (negative effect, VIP = 0.9) was a critical variable in the biorelevant model revealing that the enhanced drug solubilization in biorelevant media [51, 58] results in saturation of the powder surface with more MgSt particles when excipient level is increased. Medium characteristics (gastric or intestinal conditions) are critical on the impact of MgSt on drug apparent solubility in biorelevant media, as indicated by the significance of the variable medium (negative effect, VIP = 0.8) in the model. The presence of bile salts in high amounts in the biorelevant basic medium leads to enhanced powder wettability [51] and saturation of powder surface by excipient particles, as explained above.

The impact of MgSt variability on drug apparent solubility relates to drug physicochemical properties, as demonstrated by the significance of certain interactions in the studied models. In compendial media, CRS*solubility (negative effect, VIP = 1.2) and CRS*lipophilicity (positive effect, VIP = 0.9) were significant variables in the model indicating that the reduction in drug solubility at 24 h by the presence of crystalline MgSt brands will be more pronounced for highly soluble less lipophilic drugs. In biorelevant media, PSD*lipophilicity (negative effect, VIP = 1.1) and PSD*solubility (negative effect, VIP = 1.0) were significant interactions in the model indicating that the high particle size brands will significantly reduce the apparent solubility of highly soluble or highly lipophilic drugs. CRS*solubility (negative effect, VIP = 1.1) and CRS*lipophilicity (positive effect, VIP = 1.2) were also significant interactions in the biorelevant model revealing that the decrease in drug apparent solubility by crystalline MgSt brands will be pronounced for highly soluble hydrophilic drugs. An interplay between excipient properties was observed in compendial media by the significance of CRS*PSD (positive effect, VIP = 1.2) in the model. As MgSt brands of lower crystallinity have low lubrication efficiency, decreasing MgSt particle size will be critical only for crystalline brands leading to a higher reduction in drug solubility at 24 h compared with high PSD brands. The complexity of the impact of MgSt on drug solubility is demonstrated with the use of multivariate data analysis. The results can either be used to identify drug case studies where MgSt variability can complicate drug solubility. A more holistic approach to the impact of MgSt presence on drug solubility can be drawn with the downside that data interpretation becomes more complex due to the inclusion of different compounds in the statistical models.

### Road Map of MgSt Effects on Drug Apparent Solubility

**Fig. 8:**
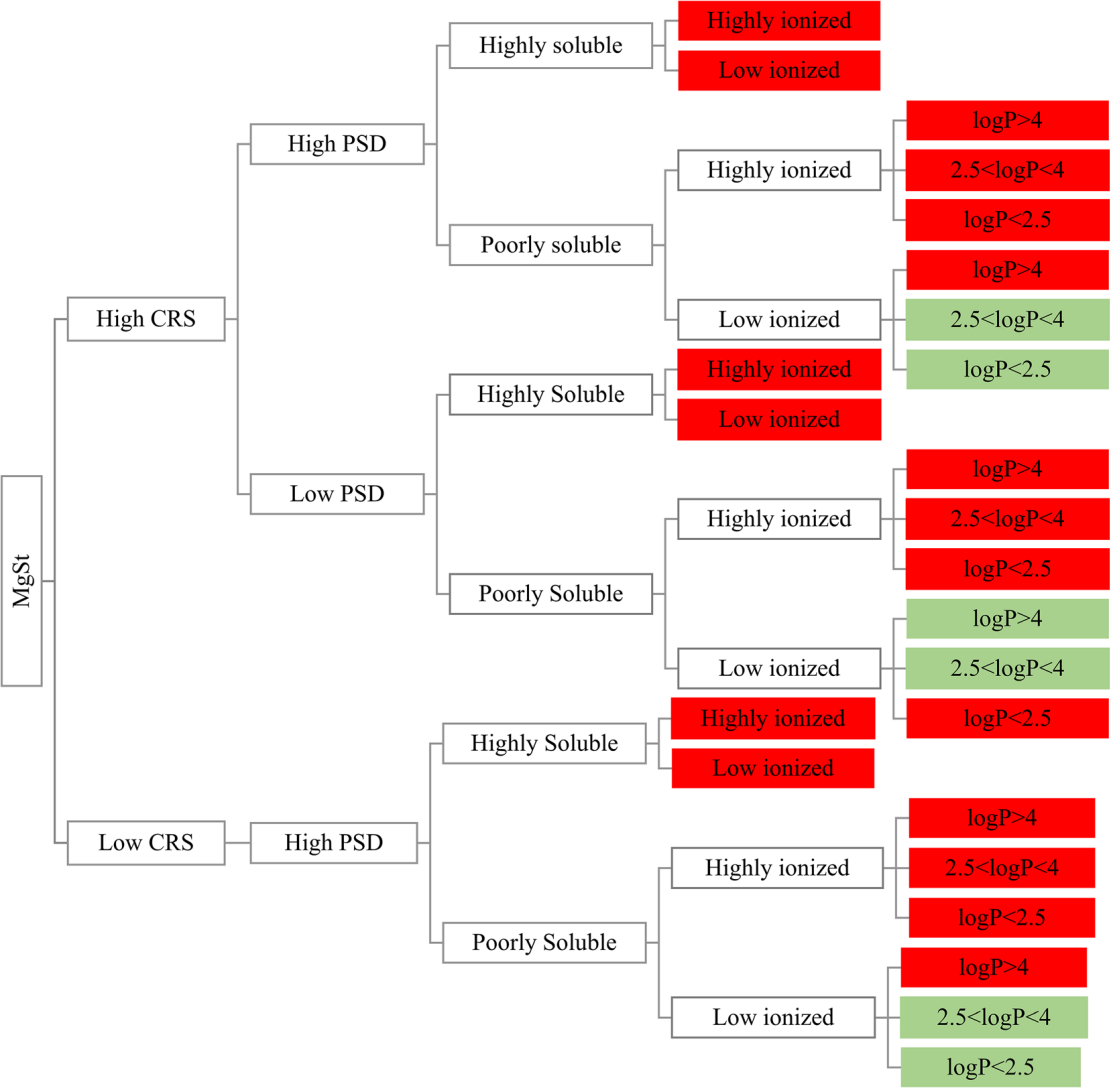
Road map of the effects of the studied excipients on drug solubility. Red boxes and green boxes indicate significant and insignificant changes in drug solubility by excipient presence, respectively

The roadmap categorizing the excipient REs on drug apparent solubility according to excipient and drug properties is presented in Fig. 8 (cases where increased drug solubility was caused by a potential shift in the pH of the solution were not considered).

The impact of MgSt on drug solubility at 24 h relates to drug physicochemical characteristics. Significant changes in drug apparent solubility by MgSt can be anticipated for highly soluble drugs, irrespective of drug ionization state (low or highly ionized). The effects of the studied MgSt brands on the apparent solubility of poorly soluble drugs depend on the state of drug ionization, as significant changes in drug solubility by MgSt presence can occur when poorly soluble drugs are highly ionized irrespective of their lipophilicity. For poorly soluble/low ionized drugs, MgSt is not expected to significantly affect drug solubility, except from the case of lipophilic drugs (log *P* > 4) in presence of MgSt brands of high PSD. The construction of the roadmap reveals that excipient selection or change needs to be thoroughly considered as excipient variability may be problematic for oral drug performance.

## CONCLUSION

The presence of certain critical excipients and changes in their critical material attributes in oral dosage forms can affect product performance. MgSt is a commonly used lubricant in immediate-release formulations but it can compromise drug dissolution. The current study focused on identifying the biopharmaceutical implications of MgSt presence and variability on drug apparent solubility. Solubility studies showed that for the majority of cases, the presence of MgSt significantly decreased drug apparent solubility due to its hydrophobic nature. The extent of the changes in drug apparent solubility depended on the material attributes of the studied MgSt brands, with excipient CRS and PSD being the most influential. The complex nature of excipient variability was revealed as the effects of excipient properties on drug apparent solubility strongly related to drug physicochemical properties (drug ionization, drug lipophilicity, drug aqueous solubility) and medium characteristics (pH/presence of bile salts). Cases where lower magnesium stearate levels may affect drug solubility will be considered for future work.

The identification of cases where MgSt presence and variability significantly affected drug apparent solubility reveals the need for assessing its impact on formulation performance. This systematic investigation demonstrates that variability in MgSt needs to be considered when making changes to drug product composition or excipient grade, as varying its properties can result in an effect on drug solubility.

## Electronic Supplementary Material

ESM 1 (DOCX 31 kb)
