# Supplementary material for: Impact of Magnesium Stearate Presence and Variability on Drug Apparent Solubility Based on Drug Physicochemical Properties
Source: AAPS J. 2020 May 21;22(4):75. doi: 10.1208/s12248-020-00449-w (PMC7242257; doi:10.1208/s12248-020-00449-w)
Supplement: Supplementary file 1 — (DOCX 31 kb) [file 12248_2020_449_MOESM1_ESM.docx]

**Supplementary Data**

**Supplementary Table I:** HPLC methods used for drug quantification

|  | **Column** | **Mobile Phase** | **Flow Rate (mL/min)** | **Temp (°C)** | **Inj. Vol. (μL)** | **Detection wavelength (nm)** | **R_t_ (min)** | **Concentration of stock solutions (mg/mL)** | **Calibration range in acidic media (μg/mL)** | **Calibration range in basic media (μg/mL)** | **Reference** |
| --- | --- | --- | --- | --- | --- | --- | --- | --- | --- | --- | --- |
| **MTF** | Inertsil Phenyl (Metachem) 250x3mm - 5μm | MeOH/Phosphate buffer pH 7 (70:30) | 1 | 20 | 20 | 236 | 8 | 2 | 10 - 200 | 10 - 200 | (1) |
| **PRC** | Spherisorb (Waters) C18 250x4.6mm - 5μm | MeOH/Water (20:80) | 1 | 20 | 20 | 257 | 6 | 2 | 10 - 200 | 10 - 200 | (2) |
| **SMX** | Polaris (Metachem) C18 250x4.6mm – 5μm | MeOH/Phosphate buffer pH 6.8 (20:80) | 1 | 25 | 20 | 257 | 7 | 1 | 10 - 200 | 50 – 500 | (3) |
| **FRS** | Spherisorb (Waters) C18 250x4.6mm - 5μm | MeOH/water with 0.1% formic acid (50:50) | 1 | 25 | 50 | 232 | 4 | 1 | 2 – 20 | 10 - 200 | (4) |
| **CBZ** | Spherisorb (Waters) C18 250x4.6mm - 5μm | MeOH/Water (60:40) | 1 | 25 | 100 | 285 | 4 | 1 | 10 – 150 | 10 - 150 | (5) |
| **DPL** | XBridge Shield C18 150x4.6mm – 3.5μm | ACN/water with 0.1% TFA (30:70) | 1 | 25 | 50 | 284 | 6 | 1 | 10 -200 | Compendial  1 - 5  Biorelevant  2 - 10 | (6) |
| **IBU** | Eclipse XDB-C18(Agilent) 250x4.6mm – 5μm | MeOH/water with 0.2% acetic acid (65:35) | 1 | 25 | 100 | 233 | 6 | 1 | 5 – 40 | 10 - 200 | (7) |
| **ITZ*** | XBridge Shield C18 150x4.6mm – 3.5μm | ACN/phosphate buffer pH 3 (60:40) | 1 | 20 | 100 | Emission: 252  Excitation: 360 | 8 | 0.1 | Compendial  0.5 – 5  Biorelevant  0.1 - 10 | 0.015 – 0.06 | (8) |

*Quantification was made using HPLC-Fluorescence. MTF = metformin, PRC = paracetamol, SMX = sulfamethoxazole, FRS = furosemide, CBZ = carbamazepine, DPL = diyridamole, IBU = ibuprofen, ITZ = itraconazole

**References**

1. Porta V, Schramm SG, Kano EK, Koono EE, Armando YP, Fukuda K, et al. HPLC-UV determination of metformin in human plasma for application in pharmacokinetics and bioequivalence studies. JPharmBiomedAnal. 2008;46(1):143-7.

2. Gao N, Qi B, Liu FJ, Fang Y, Zhou J, Jia LJ, et al. Inhibition of baicalin on metabolism of phenacetin, a probe of CYP1A2, in human liver microsomes and in rats. PLoS One. 2014;9(2):e89752.

3. Vree TB, Hekster YA, Baars AM, Damsma JE, Kleijin EV. Determination of trimethoprim and sulfamethoxazole (co-trimoxazole) in body fluids of man by means of high-performance liquid chromatography. Journal of chromatography. 1978;146(1):103-12.

4. Sora DI, Udrescu S, Albu F, David V, Medvedovici A. Analytical issues in HPLC/MS/MS simultaneous assay of furosemide, spironolactone and canrenone in human plasma samples. JPharmBiomedAnal. 2010;52(5):734-40.

5. Vertzoni MV, Reppas C, Archontaki HA. Sensitive and simple liquid chromatographic method with ultraviolet detection for the determination of nifedipine in canine plasma. Analytica Chimica Acta. 2006;573-574(Supplement C):298-304.

6. Soderlind E, Karlsson E, Carlsson A, Kong R, Lenz A, Lindborg S, et al. Simulating fasted human intestinal fluids: understanding the roles of lecithin and bile acids. MolPharm. 2010;7(5):1498-507.

7. Tan A, Eskandar NG, Rao S, Prestidge CA. First in man bioavailability and tolerability studies of a silica-lipid hybrid (Lipoceramic) formulation: a Phase I study with ibuprofen. DrugDelivTranslRes. 2014;4(3):212-21.

8. Ghazal HS, Dyas AM, Ford JL, Hutcheon GA. In vitro evaluation of the dissolution behaviour of itraconazole in bio-relevant media. IntJPharm. 2009;366(1):117-23.
